# “Ryanopathies” and RyR2 dysfunctions: can we further decipher them using in vitro human disease models?

**DOI:** 10.1038/s41419-021-04337-9

**Published:** 2021-11-01

**Authors:** Yvonne Sleiman, Alain Lacampagne, Albano C. Meli

**Affiliations:** PhyMedExp, University of Montpellier, INSERM, CNRS, Montpellier, France

**Keywords:** Ventricular tachycardia, Ventricular tachycardia

## Abstract

The regulation of intracellular calcium (Ca^2+^) homeostasis is fundamental to maintain normal functions in many cell types. The ryanodine receptor (RyR), the largest intracellular calcium release channel located on the sarco/endoplasmic reticulum (SR/ER), plays a key role in the intracellular Ca^2+^ handling. Abnormal type 2 ryanodine receptor (RyR2) function, associated to mutations (ryanopathies) or pathological remodeling, has been reported, not only in cardiac diseases, but also in neuronal and pancreatic disorders. While animal models and in vitro studies provided valuable contributions to our knowledge on RyR2 dysfunctions, the human cell models derived from patients’ cells offer new hope for improving our understanding of human clinical diseases and enrich the development of great medical advances. We here discuss the current knowledge on RyR2 dysfunctions associated with mutations and post-translational remodeling. We then reviewed the novel human cellular technologies allowing the correlation of patient’s genome with their cellular environment and providing approaches for personalized RyR-targeted therapeutics.

## Facts

- RyR2 dysfunctions are due to mutations and pathological post-translational modifications.
- Ryanopathies are not limited to cardiac diseases but rather cause a multiple-organ dysfunction.
- The hiPSC technology brings new tools to investigate ryanopathies in multiple organs in patient-specific molecular and cellular environment.

## Introduction

The ryanodine receptor is the largest membrane protein we know. It is a huge intracellular macromolecular complex formed by a homotetramer embedded in the endoplasmic reticulum (ER) and sarcoplasmic reticulum (SR). Among three isoforms, the type 2 is mostly expressed in the heart, brain, and pancreas [1]. In cardiomyocytes (CMs), the opening of the L-type calcium voltage-dependent channels causes a first entry of the extracellular Ca^2+^ that triggers the binding of Ca^2+^ to cardiac ryanodine receptor/calcium (Ca^2+^) release channel (RyR2) and allows a further release of Ca^2+^ from the SR to reach the cardiac contraction. This mechanism involving RyR2 is known as calcium-induced-calcium-release (CICR) brought by Fabiato [2]. In neurons, intracellular Ca^2+^ cycling involves the RyR and inositol [1, 3, 4]-trisphosphate receptors (IP_3_R). RyR2 expression is distributed through the central nervous system including hippocampus. RyR2 Ca^2+^ release contributes to translate synaptic activity into neuronal function [5] and stimulate learning and memory [3]. RyR2 mutations are associated to inherited disorders. The first mutations found in the RyR2, were associated to catecholaminergic polymorphic ventricular tachycardia (CPVT) [4, 6]. Today, numerous mutations of the RyR2, defined as ryanopathies, are related to some arrhythmogenic disorders including CPVT, arrhythmogenic right ventricular cardiomyopathies (ARVC) under stress conditions or short-coupled polymorphic ventricular tachycardia (VT) when at rest- [7–10]. While novel RyR2 mutations have been identified in patients, our understanding of the link between RyR2 mutations and arrhythmias has been driven by experimental models, mostly through transgenic mice and heterologous recombinant RyR2 expression systems. However, the main issue with such models remains the further transposition of identifiable mechanisms in human [11, 12]. Hence, the electrophysiological kinetic properties of a mouse heart are different from the human ones in terms of both, action potential (AP) and electrocardiogram (ECG) [13]. The murine resting heart rate is about ten times as fast as that of human. In rodents, 90–92% of the reuptake of Ca^2+^ is ensured by SR Ca²^+^-ATPase (SERCA) compared to only 76% in human. The Ca^2+^ left is extruded out of the cell via the sodium-calcium exchanger (NCX). It has been shown that the stimulation frequency can affect the relative contribution of SERCA and NCX in human but not in rat [14]. Consequently, the murine model is sensitive to evaluate the SR-dependent arrhythmias [15]. Murine CMs therefore present electrical properties that are divergent when compared to that of their human counterparts [16].

The investigation of the recombinant RyR2 proteins in vitro has been very fruitful to provide for molecular patterns. However, the cellular context and some key RyR2 partners were missing. In fact, the lab animal and in vitro recombinant models tend to oversimplify the pathological mechanisms and phenotypes observed in patients. Human models are continuously needed to improve our understanding of the RyR2 physiological and pathophysiological roles [13].

Investigating arrhythmogenic disorders by obtaining fresh ventricular cardiac biopsies from patients and healthy control subjects is both ethically and technically difficult. This is where the human stem cell technologies provide for new possibilities to resolve these limits, particularly the human-induced pluripotent stem cells (hiPSCs). The hiPSCs are self-renewable cells that are theoretically capable to differentiate into many somatic cell types [17]. They have very limited ethical issues when compared to human embryonic induced pluripotent stem cells (hESC). The hiPSC harbor the genetic background of the patients. Thus, patient-specific hiPSC-derived cardiomyocytes (hiPSC-CMs) represent a novel complementary alternative model to investigate inherited arrhythmogenic disorders associated with ryanopathies and RyR2 dysfunctions.

The present review focuses on the ryanopathies and pathological RyR2 remodeling responsible for cardiac, neuronal, and pancreatic dysfunctions as a multi-organ syndrome. We also discuss the interest of exploiting hiPSCs as a tool to model the ryanopathies in a patient-specific context to further study the genome/phenotype relationship.

## The RyR2 dysfunction and cardiac pathophysiological conditions

### Ryanopathies inducing catecholaminergic polymorphic ventricular tachycardia (CPVT)

The CPVT is an inherited cardiac syndrome that is characterized by exercise-induced VT leading to some episodic syncope. CPVT is responsible for sudden cardiac death (SCD) during exercise, acute stress and/or emotions. CPVT patients have no structural cardiac abnormalities [18–20].

CPVT syndromes have been associated with several mutated proteins in the following manner:

(i) In more than 70% of the cases, with dominant cardiac ryanodine receptor (*RYR2*) mutations (denoted CPVT1) [4, 8].
(ii) In less than 5%, with autosomal recessive or dominant calsequestrin2 (*CASQ2*) mutations (denoted CPVT2) [21]. They affect Ca^2+^ buffering in the SR and lower its binding to RyR2 [22].
(iii) Some homozygous mutations of the Trans-2,3-Enoyl-CoA Reductase Like gene (*TECRL*) are associated with both the CPVT syndrome (denoted CPVT3) and the long-QT syndrome (LQTS). They lead to an increase of the diastolic Ca^2+^ concentration and reduce the SR Ca^2+^ load, triggering then the delayed-after-depolarizations (DADs), that are responsible for these arrhythmogenic disorders [23].
(iv) Some autosomal dominant mutations of calmodulin (*CALM1*, *CALM2*, and *CALM3*) (denoted CPVT4). They perturb the CaM affinity for Ca^2+^ and RyR2 at low Ca^2+^ concentration levels, leading to diastolic SR Ca^2+^ leak [24].
(v) Some autosomal triadin gene (*TRDN*) recessive mutations (denoted as CPVT5). These provoke a total absence of this protein, further disrupting the FKBP12.6 and RyR2 interaction or either causing a reduced calsequestrin expression; calcium homeostasis in the heart is then disturbed [25, 26].
(vi) Other genes are implicated in the pathophysiological mechanism of CPVT but in far lesser extent such as ankyrin 2 (*ANK2*) [27, 28], sodium voltage-gated channel alpha subunit 5 (*SCN5A*) [29] and potassium voltage-gated Channel subfamily J member 2 (*KCNJ2*) [30–32]. However, the diagnosis of CPVT syndrome remains questionable for these cases [33].

Nowadays, over 150 RyR2 variants are associated with CPVT (see Table 1). The majority of these RyR2 variants are in four domains called hot-spot domains, well conserved between human, dog, rat, mouse, and pig.

**Table 1:** List of the RyR2 mutations associated with the CPVT syndrome.

	Localization	Mutations	Findings	Ref.
Functional characterization of the RyR2 mutants	N-terminal domain	E189D	The RyR2-E189D mutation increased the propensity for SOICR, without altering the FKBP12.6 affinity to bind to the channel.	[1]
		G230C	This novel CPVT mutation enhances RyR2 cytosolic Ca^2+^ sensitivity which leads to diastolic SR Ca^2+^ leak under stress conditions. RyR2 leak was associated with a depletion of the stabilizing FKBP12.6 protein, which eventually provoked arrhythmias.	[2]
		ΔExon 3	The *RYR2* exon 3 deletion causes a NTD alteration and results in a Ca^2+^ release properties adjustment. Although this deletion is rescued by the β strand switching, it affects interfaces with other *RYR2* domains. This suggests some N-terminal domain and channel pore coupling.	[3]
		G357S	The RyR2-G357S mutation reduced the expression of the RyR2 protein and increased the arrhythmogenic SOICR in HEK293 cells, which might be responsible for the CPVT syndrome.	[4]
		A165D	The RyR2-A165D mutation was first identified in a CPVT patient. When using a knock-in mice model, the A165D mutation induced SR Ca^2+^ release triggering DADs. The A165D mutation was located in the conformational stability loop, which explained the occurrence of some diastolic leak that is responsible for arrhythmias.	[5]
	Helical domain 1	S2246L	Increase of Ca^2+^ release in HL-1 cardiomyocytes expressing mutant hRyR2, after caffeine and β-adrenergic activation.	[6]
		P2328S	This mutation decreases FKBP12.6 binding to the RyR2. Sensitivity increases with cytosolic Ca^2+^ allowing a higher open probability of RyR2 channels at low diastolic levels, causing SR Ca^2+^ leaks in the CPVT1 syndrome. The JTV519 Rycal molecule rescued a normal RyR2 function.	[7]
		R2401H	RyR2-R2401H mutation is located in the FKBP12.6 RyR2 binding region, which could affect the CICR and the ECC resulting in a CPVT.	[8]
		S2246L, R2474S	RyR2 mutations increased both store-overload-induced Ca^2+^ release (SOICR) activity and sensitivity towards luminal calcium, without affecting the channel affinity for the FKBP12.6 in CPVT.	[9]
		N23861	The RyR2-N23861 mutation induced some sensitivity impairment towards Ca^2+^ dependent channel inhibition.	[10]
		R2267H	A novel mutation was identified in sudden infant death syndrome cases. When using some heterologous system expression, this mutation was leaky under beta-adrenergic stimulation, leading to a PKA-phosphorylation that triggers cardiac arrhythmias. Interestingly, another study demonstrated a lack of pathogenicity of this variant. Thus, the in vitro functional findings were not translated to human phenotype.	[11, 12]
		R2474S	The RyR2-R2474S mutation perturbed the interdomain conformational changes, which destabilized the closed state of the RyR2 and lead to a leaky channel.	[13, 14]
	Central domain	N4104K	See findings of the S2246L mutation.	[6]
		Q4201R	See findings of the P2328S mutation.	[7]
		Q4201R	See findings of the S2246L and R2474S mutations.	[9]
		S4153R	This novel RyR2 heterozygous mutation was first described in a 25-year-old CPVT syndrome female patient. This mutation is characterized by some RyR2 gain-of-function that is induced by the SOICR threshold reduction and some propensity increase for spontaneous calcium release.	[15, 16]
	Channel domain	R4497C	See findings of the S2246L mutation.	[6]
		V4653F	See findings of the P2328S mutation.	[7]
		I4867M,	See findings of the S2246L and R2474S mutations.	[9]
		A4860G	When using mice models and HEK293 cells, the RyR2-A4860G mutation reduced the channel activity by inhibiting Ca^2+^ release during the diastole and by overloading the SR with Ca^2+.^ Consequently, it prolonged Ca^2+^ release and corresponding AP, leading to the activation of the NCX exchanger. The I_Ti_ current triggers the early afterdepolarizations (EADs) that are responsible for CPVT pathogenesis.	[17, 18]
		S4565R	Two novel mutations were identified in sudden infant death syndrome cases. When using some heterologous system expression, these 2 mutations were leaky under beta-adrenergic stimulation, leading to a PKA-phosphorylation that triggers cardiac arrhythmias.	[11]
		R4496C (human: R4497C)	The RyR2-R4496C mutation induced an increase of the SR Ca^2+^ load responsible for Ca^2+^ waves and arrhythmias in CPVT murine model.	[19, 20]
		K4750Q	The RyR2-K4750Q mutation mediated-CPVT induced diastolic SR Ca^2+^ leak caused by an enhance of propensity to activation of cytosolic and luminal Ca^2+^ and by the loss of cytosolic Ca^2+^/Mg^2+^-mediated inactivation.	[21]
		I4855M	The RyR2-I4855M mutation was present in 2 members of a CPVT-affected family. The RyR2-I4855M shows with some loss-of-function and is characterized by some CICR inhibition of the HEK293 cells. The I4855A may interfere with Ca^2+^ permeation and may affect interactions between the RyR2 pore subunits.	[22]
Case reports and genotyping studies of patient cohorts	N-terminal domain	R414L, I419F, P164S	Novel RyR2 mutations were associated with the CPVT1 syndrome in a swimming-triggered arrhythmia syndrome using direct DNA sequencing and denaturing high-performance liquid chromatography. The 388 unrelated patients were chosen according to family or personal history of drowning or swimming related cardiac events. However, considering the large number of the cohort they did not specify the cardiac phenotype of each patient.	[23]
		ΔExon 3, A77V	In a 17-year-old boy postmortem study, the RyR2-A77V mutation was associated with both an arrhythmogenic right ventricular cardiomyopathy and a CPVT syndrome, in the same family. This 17-year-old boy presented right ventricular fibrofatty and fatty myocardium replacement and calcium phosphate deposits in right ventricular cardiomyocytes that were mostly restrained into mitochondria. His mother and his sister presented normal right and left ventricles volume and no kinetic alterations. The exercise treadmill stress test revealed polymorphic ventricular tachycardia that were successfully abolished with β-blocker (Acebutolol) treatment. The same RyR2-A77V mutation led to distinct diseases in the same family members. This reflect the complexity of clinical diagnosis and the variable phenotype that can be present even among family members of the same family. De novo *RYR2* exon 3 deletion was reported in a severe CPVT case. This patient also developed some left ventricular non-compaction (LVNC), which exacerbates the arrhythmia. This patient showed no sign of endomyocardial inflammation and displayed normal heart structure. Multiform of premature ventricular contractions, ectopic atrial rhythm and ventricular triplet were observed during exercise. She experienced ventricular fibrillation and underwent ICD implantation together with the administration of Metoprolol and then Satolol treatment. Due the severity of her phenotype, she started Flecainide and Nadolol treatment and underwent bilateral sympathectomy. The interaction between RyR2- ΔExon 3 and LVNC that may represent a predictive clinical marker for a more severe CPVT phenotype remains unclear.	[24, 25]
		R414C	The molecular autopsy revealed novel mediated CPVT syndrome RyR2 mutations in 2 unexplained drowning cases. This patient carrying the RyR2-R414C variant experienced syncope and seizure-like symptoms. Unexceptional and unremarkable EEG and physical examination were found. She was first diagnosed with acute seizure activity secondary to trauma. Due to the nature of the sudden death, direct DNA sequencing and polymerase chain reaction, denaturing high-performance liquid chromatography were performed which revealed this missense novel RyR2 mutation. As this patient presented normal structural heart and absence of fatty infiltration, she was considered as a CPVT patient.	[26]
		V186M, P164S	Four patients (3 males) out of 8 patients, were presented RyR2 mutations associated with some CPVT syndrome. Each patient presented specific symptoms which reflect the heterogeneity of CPVT phenotypes. Some patients had palpitations and seizure-like activity others cardiac arrest with ventricular fibrillation. Unfortunately, they did not match each RyR2-variant with its specific phenotype.	[27]
		R169Q	One RyR2 novel heterozygous mutation in exon 8 was screened in an 18 years old female patient presenting a CPVT syndrome. This patient presented sudden collapse due to exercise and had bidirectional ventricular tachycardia during exercise stress test. She had a good response to the β-blocker treatment. This same mutation was found recently in three unrelated females. Interestingly, all of these patients presented left ventricular non-compaction cardiomyopathy and two of them survived sudden cardiac arrest. In vitro functional analysis of this mutation revealed an increase of the Ca^2+^ fractional release from the SR and a decreased threshold for overload-induced Ca^2+^ release. It was suggested that this RyR2-R169Q mutation leads to local structural abnormalities within or near the hot-spot regions which in turn leads to functional perturbations. It leads to allosteric dysregulation by reducing the side chains size and diminishing the positive charge and stacking interaction of the RyR2 protein.	[28–30]
		L62F, M81L, P164S, E243K, F329L, R332W, V377M, G357S, T415R, R420Q, V507I, A549V, S616L, H240R	A cohort of CPVT patients was screened to investigate *RYR2* gene mutations. 34 novel mutations were identified. They did not specify the clinical phenotype of the 155 unrelated patients examined in this study. Interestingly, they proposed a novel targeted genetic testing for CPVT syndrome. They emphasized also genotype/phenotype relationship as the majority of these mutations were localized in the so-called hot-spot regions.	[31]
		D242V, E243K	The long-term follow-up of 101 CPVT patients showed high cardiac events, despite some β-blockers treatment in 21% of patients with 13% of fatal or near-fatal events. Some of these patients survived cardiac arrest and presented palpitations and syncope accompanied or not with seizures. 80% of these patients were treated with β-blockers (mostly with Nadolol but also with Propranolol, Bisoprolol, Acebutolol and Pindolol). ICD implantation and Verapamil were added to some patients after the 1st cardiac event. Even though β-blockers lower the cardiac events rate they are not sufficient alone to prevent arrhythmias.	[32]
		R169L	This mutation was identified in an 8 years old boy with CPVT and Left Ventricular Hypertrophy. This boy presented with two episodes of emotion-triggered syncope and could not survive the third one that led to sudden cardiac death. This patient carried two other mutations the G1339 variant in ATP-binding cassette, subfamily C member 9 (*ABCC9*) and R52H variant in Potassium Inwardly Rectifying Channel Subfamily J Member 5 (*KCNJ5*). These 2 variants have unknown significance. The combination between CPVT and Left Ventricular Hypertrophy might lead to a more severe fatal phenotype. However, more studies are needed to elucidate the pathophysiological mechanism underlying the structural alterations of this RyR2 mutation. This same mutation was also reported in another 9 years old girl who experienced syncopal episode. The ECG findings were not reported.	[33, 34]
	SPRY1	R739H	See findings of the L62F mutation.	[31]
	P1	R1013Q, R1051P	See findings of the L62F mutation.	[31]
	SPRY2	A1136V, T1107M,	See findings of the L62F mutation.	[31]
	Handle domain	E1724K	Independently of the localization of the RyR2 mutations, all CPVT patients presented some bradycardia and responded to the β-blockers (Nadolol, Propranolol and Metoprolol) treatment. These patients presented mono or polymorphic premature ventricular beats (MPVB/PPVB) that trigger bidirectional ventricular tachycardia and polymorphic ventricular tachycardia (PMVT) salvos. 9-year-old was the median age of symptoms onset. The proband carrying the RyR2-E1724K mutation presented monomorphic bigeminy (BG) and PMVT upon exercise stress test.	[35]
		E1837K, E2045G	See findings of the L62F mutation.	[31]
		V1810L	A novel CPVT syndrome associated RyR2 mutation was identified during the screening of 35 Kazakhstani patients. This low-penetrance variant was found in a 42-year-old Korean proband. Initially, this patient was diagnosed with idiopathic arrhythmia characterized by unstable paroxysms of ventricular tachycardia. He presented bigeminy with a sinus rate of 83 bpm and reached 220 bpm during VT which was monomorphic.	[36]
	Helical domain 1	S2246L, R2474S	Priori’s group was the first who reported a direct relationship between RyR2 missense variants and CPVT syndrome. 4 missense mutations have been identified including 3 de novo. The RyR2-S2246L variant was identified in an 8-year-old boy who presented spontaneous onset of bidirectional VT upon isoproterenol infusion. Nadolol and ICD implantation proved effective for this proband. The RyR2-R2474S variant was also found in an 8-year-old boy who presented non-sustained bidirectional VT upon exercise stress test. He was treated with Atenolol.	[37, 38]
		P2328S	Missense RyR2 gene mutation was identified in CPVT patients, which could affect the myocardial calcium signaling.	[39]
		R2311D, E2311D	The arrhythmogenic events occurred in young RyR2 mutations-affected patients, when compared to ungenotyped CPVT patients, with a higher risk of syncope for males.	[40]
		V2306I, P2328S	Novel mutations were found to be associated with the CPVT syndrome in 12 Finnish probands.	[41]
		A2387P	Novel RyR2 mutation was screened and identified using the DHPLC approach.	[42]
		A2403T	See findings of the R414L mutation.	[23]
		L2487I	RyR2 mutation was detected in 6% of unrelated genotype negative and atypical LQTS, that was considered as CPVT patients.	[43]
		A2254V, A2394G	Independently of the localization of the RyR2 mutations, all CPVT patients presented some bradycardia and responded to the β-blockers treatment. 9-year-old was the median age of symptoms onset. The proband carrying the RyR2-A2254V mutation survived cardiac arrest (CA) and presented BG and polymorphic couplets (PC) upon exercise stress test. Whereas, the patient carrying the RyR2-A2394G mutation presented with seizures during the syncopal events and survived CA. Her exercise stress test revealed MPVB and PMVT.	[35]
		V2475F	The molecular autopsy revealed novel mediated CPVT syndrome RyR2 mutations in 2 unexplained drowning cases. The boy had negative toxicology screen results and no sign for trauma and structural cardiovascular abnormalities. Direct DNA sequencing revealed the presence of this novel RyR2-V2475F variant.	[26]
		R2359Q	Novel RyR2 mutations was identified in 2 CPVT families. The ECG performed for 3 patients from these families, revealed U-wave alterations	[44]
		L2534V	A 13-year-old boy case study, with some novel RyR2 heterozygous mutation. An implantable recording loop was used to diagnose arrhythmogenic disorders.	[45]
		R2404T	Some RyR2 novel heterozygous mutations were showed to be associated with a CPVT syndrome, in a family exhibiting some long QT-syndrome.	[46]
		F2307L	Genetic screening for long QT and CPVT syndrome patients in Norway.	[47]
		V2113M, Y2156C, H2168Q, E2183V, D2216V, E2296Q, F2307L, V2321M, R2404T, R2420W, M2389L	See findings of the L62F mutation.	[31]
		H2217Y, C2402Y	See findings of the D242V mutation.	[32]
		G2337V	The β-blockers treatment suppressed severe arrhythmias in stress-induced CPVT related RyR2 mutations, though it did not prevent the less severe ones.	[48]
		L2527W	Determination of a novel RyR2 heterozygous mutation in a 9-year-old Chinese boy, misdiagnosed with epilepsy and CPVT syndrome. The β-blocker (Metoprolol) treatment proved unfavorable.	[49]
		E2296K	This RyR2-E2296K mutation was identified in a 5-year-old Chinese boy with CPVT using the whole exome sequencing. This mutation might reduce the protein stability. However, further investigations are needed to prove its causality.	[50]
		V2193L	The RyR2-V2193L mutation was identified in a 9-year-old Chinese boy who presented with both epilepsy and CPVT syndrome. The exercise stress test revealed frequent PPVB and PMVT with the presence of R on T. His electroencephalogram (EEG) showed frequent epileptiform discharges during stage II, stage III and REM sleep. He was successfully treated with Metoprolol and Levetiracetam.	[51]
		C2277R	The RyR2-C2277R variant, located in the calstabin-binding domain, was identified in 8 members of the same family. The proband and her other family members presented ventricular extrasystoles (VE), bigeminy and/or trigeminy, doublets and non-sustained VT upon exercise stress test and adrenaline test. These patients showed similar response but different ventricular arrhythmias complexity degrees. The proband was treated with the combination of ICD implantation, Flecainide and Nadolol. The other family members were treated either with Atenolol, or Nadolol or with the combination of Nadolol and Flecainide or Atenolol and Flecainide which proved effective.	[52]
		G3037D	Identification of a novel RyR2 heterozygous mutation in a 2 years old patient exhibiting some CPVT syndrome.	[53]
	Helical domain 2	N4104K	See findings of the mutation S2246L. The RyR2-N4104K variant was identified in a 14-year-old boy who presented non-sustained bidirectional VT upon exercise stress test. This proband was efficiently treated with Atenolol.	[37, 38]
	Central domain	Q4201R	Missense RyR2 gene mutation was identified in CPVT patients, which could affect the myocardial calcium signaling.	[39]
		L3778F, G3946S	See findings of the R2311D mutation.	[40, 54]
		N4097S, E4146K, T4158P	In a postmortem genetic testing model, 3 novel mutations were identified in 7 cases of sudden unexplained death, that might potentially cause CPVT.	[55]
		F4020L, E4076K, N4104I, H4108N, H4108Q	Independently of the localization of the RyR2 mutations, all CPVT patients presented some bradycardia and responded to the β-blockers treatment. 9-year-old was the median age of symptoms onset. The proband carrying the RyR2-F4020L mutation presented with seizures during the syncopal events. His exercise stress test revealed BG, PC, and PMVT. Unfortunately, he died suddenly at the age of 20. The proband carrying the RyR2-E4076K mutation presented BG and PMVT upon exercise stress test. The patient carrying the RyR2-N4104I mutation presented with seizures during the syncopal events. His exercise stress test revealed PPVB and sustained PMVT. The proband carrying the RyR2-H4108N mutation survived CA and presented BG, PC, and PMVT upon exercise stress test. Whereas, the patient carrying the RyR2-H4108Q mutation presented MPVB and PMVT upon exercise stress test. The symptoms of these patients reflect the complexity and the variability of the clinical phenotype of CPVT patients which allowed the assessment of a genotype-phenotype correlation.	[35]
		S3938R, T4196A,	See findings of the V186M mutation.	[27]
		L4105F	Novel mutation of the RyR2 mediated CPVT syndrome in 21 years old male. A β-blocker (Metoprolol) and calcium channel blocker (Verapamil) treatment, combined with the successful placement of a dual-chamber implantable cardioverter defibrillator proved effective.	[56]
		R4144C	See findings of the F2307L mutation.	[47]
		L3879P, Q3925E, G3946A, S3959L, M3972I, D3973H, L3974Q, K3997E, S4124G, Y4149s, R4157Q, Q4159P, N4178S, E4187Q	See findings of the L62F mutation.	[31]
		S3799P, G3946D, D3977Y, A4091V, A4091T	See findings of the D242V mutation.	[32]
		F4174L	A novel heterozygous mutation of the *RYR2* gene associated CPVT syndrome was identified in a 17-year-old Caucasian boy. Interestingly, arrhythmias had occurred both at rest and under sympatho-adrenergic stimulation conditions.	[57]
		A4282V, R4307C, G4315E	See findings of the L62F mutation.	[31]
	Unspecified domain	K4392R	Case report of an athlete woman harboring some gain-of-function RyR2-K4392R mutation associated CPVT syndrome.	[58]
		R4497C	See findings of the mutation S2246L. The RyR2-R4497C variant was identified in a 30-year-old female who presented non-sustained bidirectional polymorphic VT upon exercise stress test. Two of her sisters died suddenly at the age of 14 and 16 respectively. Variable age-related manifestation of the disease has been thus suggested. This proband was treated with ICD implantation.	[37, 38]
	Channel domain	V4653F	Missense RyR2 gene mutation was identified in CPVT patients, which could affect the myocardial calcium signaling.	[39]
		V4771I, A4860G, I4867M, N4895D, E4950K	See findings of the R2311D mutation.	[40]
		P4902L, R4959Q	Three novel mutations were found to be associated with the CPVT syndrome in 12 Finnish probands.	[41]
		N4504I, A4608P, V4880A, M4504I, A4607P	Four novel RyR2 mutations were screened and identified using the DHPLC approach.	[42]
		F4499C, A4510T, G4671R, I4848V	See findings of the R414L mutation.	[23]
		A4556T, 4657–4658EYinsertion, G4671R	RyR2 mutations were detected in 6% of unrelated genotype negative and atypical LQTS, that were considered as CPVT patients.	[43]
		G4662S, H4762P, P4902S	Independently of the localization of the RyR2 mutations, all CPVT patients presented some bradycardia and responded to the β-blockers treatment. 9-year-old was the median age of symptoms onset. The probands carrying the RyR2-G4662S and RyR2-H4762P mutations presented BG and PMVT upon exercise stress test. The patient carrying the RyR2-P4902S presented PPVB and PMVT upon exercise stress test.	[35]
		R4959Q	This mutation was identified in 11 patients of the same family. Four patients were diagnosed with bidirectional tachycardia. Five patients presented monomorphic ventricular tachycardia. Two patients died suddenly while asleep.	[59]
		F4851C, N4895D	Two novel RyR2 mutations were identified in 2 CPVT families. The ECG performed for 3 patients from these families, revealed U-wave alterations	[44]
		F4511L	See findings of the R2404T mutation.	[46]
		E4431K, E4611K	Genetic screening for long QT and CPVT syndrome patients in Norwegia.	[47]
		S4565R, E4611K, W4645R, K4650E, N4736 Del, R4790Q, K4805R, R4822H, G4936R	See findings of the L62F mutation.	[31]
		F4851L	See findings of the D242V mutation.	[32]
		G4671V	See findings of the G2337V mutation.	[48]
		D4631V	A novel CPVT syndrome associated RyR2 mutation was identified during the screening of 35 Kazakhstani patients. This de-novo missense variant was identified in a 23-year-old female Kazakh. 13-year-old was the age of symptom onset. She experienced syncopal episodes and MPVB/PPVB that trigger bidirectional ventricular tachycardia and PMVT salvos. Since childhood this patient suffered from dizziness, frequent respiratory infections, scoliosis, palpitation, and chronic pyelonephritis along with the CPVT syndrome. She underwent ICD implantation together with the administration of β-blockers treatment.	[36]

**Fig. 1:**
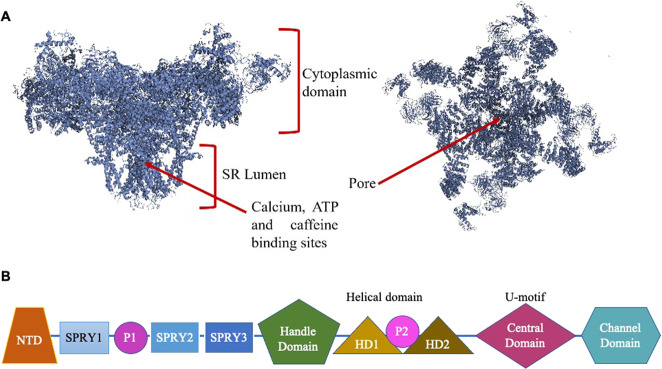
Representative 3D structure of RyR2. **A** Illustration of the 3D structure of RyR2 viewed from the cytoplasmic side and adapted from Peng et al. [35]. **B** Domain organization of the RyR2. NTD N-terminal domain, SPRY splA kinase and RyR domain, P1/P2 RyR repeat domain rich in phosphorylation sites, HD1/HD2 Helical domain.

Thanks to the available 3D RyR1 and 2 structures, we now know that the majority of the RyR2 related CPVT mutations are located in domains involved in channel activation and gating including the pore, pseudo-voltage sensor and central domains (Fig. 1) [1, 34, 35]. A potential link between mutation localization and phenotype severity has been emphasized [36], in particular mutations in the C-terminal hot-spot domain being of greater risk to patients. Mutations in the bridging solenoid and the N-terminal domains, which form the RyR2 interprotomer contact domain, influence the cytoplasmic shell dynamics: they affect the outward shell movement that is necessary for the channel pore opening. An altered RyR2 channel gating mechanism via pore opening facilitation has been suggested for elevated frequency of spontaneous Ca^2+^ sparks under-stress [1]. Mutations in the C-terminal domain directly promote the activation of the channel either by facilitating the binding of Ca^2+^ and ATP or restraining the channel inactivation. It should be noted that FKBP12.6 binds to the bridging solenoid, stabilizing the interaction between the cytoplasmic region and the pore of the channel.

The FKBP12.6 binding has not been always shown affected by CPVT-linked ryanopathies. However, ryanopathies trigger Ca^2+^ leak in any case [37, 38]. The so-called store-overload-induced calcium release (SOICR) hypothesis suggests that the CPVT-linked RyR2 mutations increase both, the sensitivity of the channel to luminal calcium and SOICR activity resulting in SR Ca^2+^ leak, DADs and arrhythmias, without affecting the affinity of the channel to FKBP12.6 [39]. Another group has demonstrated that the mitochondrial ROS induced by the Ca^2+^ leak exacerbate the RyR2 activity in a proarrhythmic feedback cycle in CPVT mouse model [40].

RyR2 homozygous multi-exon duplication was identified in 2 large Amish family with unexplained sudden deaths. Highly penetrant *RYR2* homozygous duplication, exons 1 through 4 of *RYR2* and *RYR2*’s 5′UTR/promoter region, was the main cause of death of these young subjects. This *RYR2* homozygous duplication might result in a loss of function of the channel however the pathophysiological mechanism related to the SCD has not been elucidated so far [41].

Phenotypic relationship in CPVT individuals by classifying and correlating the *RYR2* missense variants has been evaluated. The authors found that CPVT-associated *RYR2* variants predominantly occur at more conserved amino-acid positions located between amino-acid positions 3949–4332 and 4867–4967. These variants were also located in RyR and IP_3_R homology-associated and ion transport domains. However, the *RYR2* variants associated with sudden death during sleep were exclusively located in the C-terminal domain [42].

**Fig. 2:**
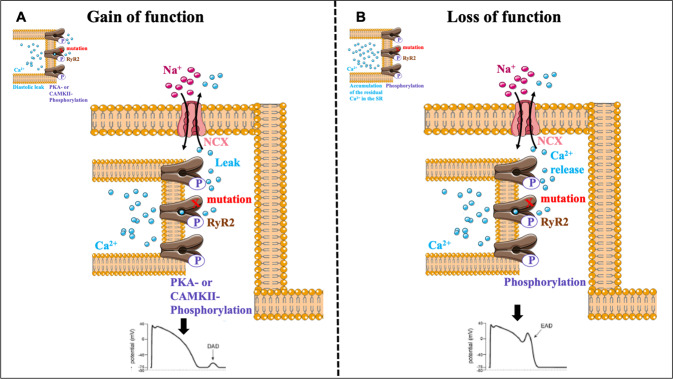
Representative diagram illustrating the consequences of RyR2 gain-of-function or loss-of-function heterozygous mutations. **A** During diastole, the PKA- or the CaMKII-phosphorylation of the RyR2 gain-of-function mutant channels induces a SR Ca^2+^ leak that increases the diastolic Ca^2+^ concentration and activates the NCX exchanger. This leads to I_Ti_ current which generates DADs and arrhythmias. **B** During AP, the I_CaL_ activates the phosphorylated RyR2-WT channels that generate a normal calcium transient with a lower amplitude which in consequences, induces an accumulation of the residual Ca^2+^ in the SR which gradually increases. When the SR Ca^2+^ load achieves the threshold of stimulation of the phosphorylated RyR2 loss-of-function mutant channels, it releases Ca^2+^. This in turn activates the neighboring RyR2-WT enhancing thus the CICR mechanism. Hence, the Ca^2+^ release through the RyR2 loss-of-function mutant channels at the end of the Ca^2+^ transient induces a second phase of Ca^2+^ release. It activates the NCX exchanger leading to I_Ti_ current which generates EADs responsible of the pathogenesis of the CPVT syndrome.

Finally, ryanopathies lead, in the majority of the cases, to gain of function (GOF) of the channel and in a lesser extent to a loss of function (LOF) (Fig. 2).

### Ryanopathies and short-coupled polymorphic ventricular tachycardia (SC-PMVT)

The Torsade de Pointes (TdP), also called ‘twisting peaks’, are cardiac rhythm disorders. These sometimes revert back into sinus rhythm or either degenerate into ventricular fibrillations (VF), syncope, and SCDs [10]. The ECG performed shows the typical TdP initiating-mode with the presence of a short-long-short sequence pattern [43, 44]. Short-coupled TdP (SC-TdP) and polymorphic ventricular tachycardia (PMVT) occur in patients with normal structural heart, in the absence of QT prolongation, myocardial ischemia and stress-induced arrhythmia exercise tests [45, 46]. They entail, in 8% of cases, the SCD of patients [10].

We first reported the existing link between a novel heterozygous mutation, RyR2-H29D, with a clinical phenotype of short-coupled PMVT at rest [10]. A RyR2-H29D mutation-harboring by mother and daughter, suffered a syncope and a short-coupled PMVT at rest. We used a heterologous recombinant system and compared the functional and molecular RyR2-H29D properties to control channels in presence of the FKBP12.6. RyR2-H29D leads to leaky RyR2 channel, at low diastolic Ca^2+^ levels, under non-stressful conditions and along with FKBP12.6 depletion. These results were challenged by Chen’s group by introducing the same variant into the RyR2 mouse sequence [47]. They could not evidence any changes in RyR2 open probability, [^3^H] ryanodine RyR2 binding, caffeine sensitivity and spontaneous Ca^2+^ release. The controversial conclusions of both studies are yet unexplained but might be related to some experimental conditions.

Other studies carried out identified novel RyR2 mutations entailing the occurrence of the SC-TdP. The RyR2-M995V variant, located in the cytoplasmic loop, outside the hot-spot region primary amino-acid sequence, was identified in a SC-TdP patient who experienced many syncopes [48]. This particular patient had a family history of sudden death and both her brother and her father carry the same RyR2-M995V variant. Despite the lack of functional characterization in this study, the co-segregation of the variant in the family suggests its involvement in the SC-TdP observed in the patient. Another study reported non-cited RyR2 variant, but its pathogenicity was studied in case of idiopathic VF case. The proband survived a SCD episode and recovered from a short-coupled extrasystole degenerating into TdP [49]. Moreover, the *RYR2* gene sequencing in 7 SC-TdP patients revealed three novel heterozygous mutations (RyR2-S4938F, -V1024I, and -A2673V) and 1 polymorphism (RyR2-N1551S) that were all located inside the cytoplasmic domain. In this study, researchers found that the RyR2-S4938F exhibited a loss of function associated with higher ER Ca^2+^ signals, [^3^H] ryanodine binding reduction to the channel and Ca^2+^ release decrease. In contrast, the RyR2-N1551S, -V1024I, and -A2673V variants demonstrated some gain-of-function properties [9]. These results suggest that, similarly to CPVT, PMVT-related RyR2 mutations are associated with both some gain- and loss-of-function. Interestingly, a novel reported heterozygous mutation RyR2-F4174I in a 17 years old Caucasian patient caused polymorphic VT both at rest and under stress conditions. This mutation may cause arrhythmia independently of the sympatho-adrenergic stimulation via unclear pathophysiological mechanism [50].

Two LOF mutations were recently identified. The RyR2-E4146K and -G4935R were associated with Ca^2+^ release deficiency syndrome and IVF. Using recombinant expression in HEK cells, Zhong et al. found that the RyR2-G4935R suppresses completely the [^3^H] RyR2 binding and the RyR2 caffeine activation quasi-similar to the RyR2-E4146K. In addition, the cytosolic Ca^2+^ activation, SOICR activity and RyR2 sensitivity to luminal Ca^2+^ were abolished in RyR2-E4146K. To compare with CPVT RyR2 GOF mutations, RyR2-E4146K and -G4935R mutations induce cardiac arrhythmias under non-stressful conditions and are silent under exercise stress testing. Therefore, RyR2 LOF mutations are associated with a distinct phenotype than CPVT and constitute a new entity of cardiac arrythmias [51].

It is noteworthy that the overexpression of the *DPP6* gene enhances the I_to_ current in Purkinje fibers, resulting in arrhythmias in IVF patients [52]. Other groups identified GOF mutations of KCNE5 which enhances the I_to_ current leading to AP shortening of Purkinje cells in IVF [53]. Moreover, loss of function mutations of the His-Purkinje system transcription factor IRX3 were identified to induce arrhythmias in IVF mouse model [54].

### Ryanopathies and arrhythmogenic right ventricular cardiomyopathy (ARVC)

Arrhythmogenic right ventricular dysplasia is also known as the arrhythmogenic right ventricular cardiomyopathy (ARVD/ARVC). This is an inherited cardiac muscle disease characterized by the replacement of the CM by fatty or fibro-fatty tissues that further means its degeneration [55]. ARVD occurs most especially in the right ventricle and provokes some abnormal contraction and ventricle dilatation with posterior ventricular arrhythmias and SCD [56]. In fifty percent of cases, the ARVD is caused by some autosomal dominant mutation in gene encoding desmosomal proteins like desmoplakin *(DSP)*, plakoglobin *(JUP)*, plakophilin-2 *(PKP2)*, desmoglein-2 *(DSG2)* and desmocollin-2 *(DSC2)* [57]. In fact, the desmosome plays a key role in maintaining the integrity of the cell membranes of the heart. Desmosomes are intercellular cell-to-cell junctions that provide a strong adhesion of the myocardium cells, enabling them to resist the mechanical stress induced during contraction itself [58].

Recessive mutations were also reported in other studies but were mainly associated with skin disorders. Other ARVD forms are often associated with mutations of the transforming beta-3 growth factor regulator (*TGFβ3*) inducing ARVD1 [59], transmembrane protein 43 (*TMEM43*) [60], desmin [61], lamin A/C [62], striatin [63], and titin [64].

The arrhythmogenic right ventricular cardiomyopathy type 2 (ARVC/D2) differs from other ARVD forms because it is a stress-induced disorder responsible for the occurrence of VT [65]. Interestingly, dominant mutations of the RyR2 were reported in the ARVD2 pathophysiological mechanism but in a far lesser extent [66]. In 2001, Tiso et al. identified four missense RyR2 mutations: RyR2-R176Q, -L433P, -N2386I, and -T2504M. These were reported to be in a critical domain for maintaining normal Ca^2+^ channel regulations and were associated with ARVD2 in Italians patients. RyR2-N2386I and -T2504M are located in the FKBP12.6 interaction domain. RyR2-R176Q and -L433P are situated in the cytosolic domain. Under β-adrenergic stimulation, these mutations induce decrease affinity of the FKBP12.6 for RyR2, some hypersensitivity of the RyR2 channel leading to a SR Ca^2+^ leak, that triggers, not only arrhythmias that are prevented with the use of β-blockers, but also some apoptosis and/or cell necrosis, which are responsible for the degenerative pathways observed in ARVD2 [67–69]. It is noteworthy that the RyR2-L433P mutation is the only one to induce a LOF resulting in a decrease of response to the caffeine activation of RyR2. The dantrolene treatment proved effective. One explanation given by the authors to this is that dantrolene restores the inter-subunit interaction of the RyR2 N-terminal domain and normal channel functions [70]. Other studies demonstrated the implication of RyR2 in ARVD2 pathogenesis, and highlighted other RyR2 mutations like RyR2-R420W, -Y2392C, and -A77V in Italian patients treated with β-blockers, that seemed to prevent the Ca^2+^ homeostasis in-balance [71]. Moreover, a German group identified 2 single nucleotide RyR2 polymorphisms, RyR2-G1885E and -G1886S in a cohort of 85 ARVC patients. When present in a *trans* configuration, they induced an abnormal calcium homeostasis and SR Ca^2+^ leak responsible for the arrhythmogenic disorders observed in these patients [72]. In a canine model (Boxer dogs) of ARVC2, lower RyR2 proteins and mRNA levels were reported in all heart chambers, with the lowest expression in the right ventricle. In the same model, other group demonstrated a depletion of the FKBP12.6 that led eventually to SR Ca^2+^ leak, which together could explain the pathophysiological mechanisms lying behind the ARVC2 syndrome [73]. When screening the *RYR2* gene in a cohort of 64 ARVC/D patients without mutation in desmosomes genes, Roux-Buisson et al. identified 6 missense rare variants: RyR2- P1583S, -A2213S, -G2367R, -Y2932H, -V3219M, and -L4670V- that were associated with a conventional phenotype of ARVC/D [74]. Interestingly, it was reported that the RyR2-T1107M mutation is, not only associated with CPVT syndrome, but also with hypertrophic cardiomyopathy (HCM) in a patient who suffered severe left ventricular outflow tract obstruction and VF [75]. HEK293 cells expressing the corresponding murine mutant, RyR2-A1107M, exhibited diminished fractional Ca^2+^ release and increased luminal Ca^2+^ threshold release termination which could reflect the HCM pathophysiological mechanism [76].

An isoform-specific effect on RyR structure was revealed by evaluating the effect of the RyR2-R176Q mutant involved in CPVT and ARVD2, and its homologous the skeletal muscle RyR1-R163C, implicated in malignant hyperthermia (MH) and central core disease. Both mutations are located in the N-terminal domain. Despite the equivalent position in 3D structure and sequence of the positive charge loss, the RyR1-R163C mutant exhibited a preactivated altered conformation and displayed salt bridge network distortion, Ca^2+^ binding site alteration, rotation of a cytoplasmic domain induced by a molecular latch and partial progression to channel open state [77].

## Ryanopathies as a multiple-organ dysfunction syndrome

### Ryanopathies and neuronal disorders

The brain regionally expresses the three RyR isoforms. The RyR2 is predominant in the cerebral cortex and in dental gyrus of the hippocampus [78]. The role of RyR2 in neuronal function and behavior has been investigated in the last decade. It has been shown that nicotine induces RyR2 upregulation via activation of the transcription factor CREB in the cortex and ventral midbrain. This upregulation induces itself a phosphorylation of CREB leading to positive feedback signaling loop [79]. Downregulation of RyR2 induces spatial memory defects while the brain-derived neurotrophic factor induces RyR2 upregulation through ROS generation which is crucial for structural plasticity [80].

Neuronal consequences of ryanopathies have been reported; Lehnart et al. showed a link between CPVT syndrome and independent tonic-clonic seizures in the CPVT RyR2-R2474S transgenic murine model. This mutation induced a Ca^2+^ leak through the RyR2, that was responsible for both arrhythmia and epilepsy episodes under stress. Both disorders were prevented by stabilizing the RyR2 closed state conformation using the Rycal S107 compound [81]. A study also evaluated the RyR2 involvement in epilepsy for CPVT patients [82]. Another recent study showed the existing link between the RyR2-A77T mutation and a generalized epilepsy in a 32 years old woman presenting no anterior cardiac manifestations. However, her brother carrying the same mutation, was diagnosed with a CPVT syndrome and survived a cardiac arrest [83].

Furthermore, the role of RyR2 in cerebral ischemia pathogenesis was reported. Increased RyR2 S-glutathionylation proved to actually enhance the CICR, resulting in an amplification of the Ca^2+^ entry and leading to further cortical neuronal death [84]. RyR2 was also reported to be involved in memory impairment. In a mouse model of stress-induced cognitive dysfunction, neuronal RyR2 was shown to be remodeled by oxidation, S-nitrosylation, PKA-phosphorylation, and depletion of FKBP12.6 and leaky. Ca^2+^ fixing with the administration of Rycal compounds or prevention of PKA phosphorylation with genetic ablation of serine 2808, rescued the cognitive dysfunction [85]. Our group also attested the existing connection between neuronal RyR2 dysfunctions and neurodegenerative disorders, notably the occurrence of the Alzheimer disease (AD). In sporadic-AD-human-patient-brain samples and in two familial AD murine models, the “biochemical signature” of the leaky RyR2 with PKA-phosphorylation -FKBP12.6 depletion was associated with an ER Ca^2+^ leak, through increased RyR2 open probability and a loss of memory [86]. Preventing the RyR2 PKA-phosphorylation on the Ser2808 site by crossing AD mice with *S2808A*^+/+^ mice improved the cognitive behavior, while *S2808D*^+/+^ mice, which harbor constitutively some PKA-phosphorylated RyR2, exhibit early cognitive and synaptic dysfunctions. These results strongly support the role played by the RyR2 PKA phosphorylation and the β-adrenergic receptor signaling cascade in AD patterns. An interplay between the β-adrenergic signaling pathway, the amyloid β (Aβ) and the altered Ca^2+^ homeostasis through the leaky RyR2 channels in AD, was also observed [87, 88]. Altered Ca^2+^ signaling via the RyR2 channels in the pathogenesis of AD was highlighted in the literature by other groups too [89–91]. Recently, Chen’s group demonstrated a hyperactivity-directed strategy to counter the progression of AD despite the continuous β-amyloid accumulation. Genetically limiting the open time of the RyR2 through the RyR2-E4872Q mutation prohibits the learning and memory alteration, intrinsic membrane hyperexcitability, neuronal hyperactivity and neuronal cell death in AD mouse model. It enhances as well the A-type K^+^ current that drive neuronal excitability. These results were confirmed with the use of a pharmacological RyR2 open time limiting, the R-carvedilol enantiomer [92]. Moreover, loss of RyR2 induces dendritic spine structural plasticity impairment during memory acquisition. Dendritic sparsification, loss of excitatory synapses and over compensatory excitability were also observed. These alterations suggest a crucial role of RyR2 in neurodegenerative diseases [93].

These reports clearly demonstrated that similar ryanopathies can affect the heart and brain organs independently. However, these results also underlined the complex interpretation of such disorders, with the same family mutation triggering different effects: CPVT and/or epilepsy. As an 11 years old boy carrying the RyR2-R2401H mutation was long-term diagnosed with epilepsy instead of CPVT [94]. This misdiagnosis was common for CPVT syndrome [95]. Thereby, this ambivalence appears to be patient-specific and therefore, the use of hiPSC biotechnology constitutes a novel tool to model these neuro-cardiac disorders associated with ryanopathies.

### Ryanopathies and metabolic syndromes

Diabetes mellitus is a chronic disease characterized by high blood sugar levels, called hyperglycemia. The glucose intolerance in these patients is due to: (i) abnormal secretion of insulin from the Langerhans cell islet located in the pancreas and induced by an autoimmune destruction of β-pancreatic cells that causes a lack of the insulin secretion usually called type I diabetes, (ii) resistance of target cells to the action of insulin inducing resistance, denoted as type II diabetes, or (iii) combination of both mechanisms [96, 97].

Insulin secretion is stimulated by glucose level elevation. Basically, glucose incorporating the β-pancreatic cells causes an increase in ATP production and induces the depolarization of the cellular membrane by inactivating the ATP-sensitive K^+^ channels. This is followed by the calcium entry through the Ca^2+^ voltage-dependent channels that further triggers a huge release of Ca^2+^ from the ER. In this mechanism, the precise role of the RyR2 in regulating the insulin secretion from the β-pancreatic cells is not fully understood. Dixit et al. demonstrated that the RyR2 GOF by constitutive CaMKII hyperphosphorylation at Ser2814 contributes to basal RyR2 ER leak, glucose intolerance, impaired glucose-stimulated insulin secretion, which constitute pre-diabetes features [98]. Is the RyR2 GOF causing glucose intolerance and promoting diabetes?

Santulli et al. brought an answer to that question by investigating the β-pancreatic cells of CPVT patients harboring RyR2 mutations. They found that CPVT RyR2 mutated patients as well as transgenic CPVT mice exhibited glucose intolerance and impaired glucose homeostasis. They also found that CPVT RyR2 channels in the pancreas were oxidized and S-nitrosylated, which activated an ER stress response, some mitochondrial dysfunction and decreased fuel-stimulated insulin release [99]. By preventing the ER Ca^2+^ leak pharmacologically, the closed conformation of RyR2 prevents these metabolic abnormalities in β-pancreatic cells.

Other groups have also investigated the role of RyR2 in insulin secretion patterns in diabetes mellitus [100, 101]. Our group demonstrated an altered Ca^2+^ homeostasis that included increased frequency of Ca^2+^ sparks, reduced amplitude of Ca^2+^ transients and depressed SR Ca^2+^ load in diabetic rats [102]. These modifications were associated with RyR2 PKA-hyperphosphorylation and a depletion of the FKBP12.6 protein. The possibility of differentiating hiPSC in insulin-producing ß-like cells [103] also opens interesting perspectives in this field of research.

## Ryanopathies modeled through human stem cell technologies

The knowledge acquired in cardiac physiology and physiopathology has been significant thanks to the animal models used [104]. However, the animal models do not always recapitulate the phenotypes observed in patients. Investigating stress consequences in murine CMs, with electrical pacing is practically impossible, because 10 Hz pacing is too fast and leads to cell death. Actually, imposing some 1 or 2 Hz electrical pacing in mouse or rat CMs is so far away from the usual physiological rodent heart rate, that it does not account for such intended stress conditions. From a genomic point-of-view, the mouse response differs from the human one in several diseases investigated [105]. The use of complementary tools and models, beside that of the mouse, in the cardiovascular research field is therefore quite necessary.

The hiPSCs were first introduced in 2007, when Yamanaka’s group succeeded at reprogramming somatic cells- the human dermal fibroblasts-, into hiPSCs, by overexpressing four defined growth factors (Oct3/4, Sox2, c-Myc, and KLF4). These are now referred as Yamanaka’s reprogramming factors [106]. These generated hiPSCs actually exhibit the main properties of hESC, including their proliferation ability, morphology, pluripotency markers, telomerase activity, epigenetic and gene expressions. They show an incredible potential to differentiate into the three germ layers and also abolish all ethical issues arising with the use of hESC [17, 106]. After these results, several groups were able to reprogram other somatic cell types into hiPSCs, including peripheral blood cells, keratinocytes, dental pulp, and urine-derived cells [107–112].

With the hiPSCs, researchers use patient-specific cells for their potential for self-renewal; these can be theoretically differentiated into all somatic cell types. The generation of hiPSC-CMs is therefore of growing interest because of its multiple applications. It can be used, first of all, to elucidate important underlying cardiac disease-driving molecular mechanisms and test novel therapies [17, 113, 114]. Secondly, the access to an in vitro human development model enhances the study of human heart patterns that would not otherwise be possible. Thirdly, the stem cell derived CMs are used as a human cardiac model to research on diverse basic questions ranging from cellular electrophysiology to protein biochemistry. Furthermore, the ability to generate hiPSCs, from patients with inherited cardiac disease, provides unprecedented opportunities to study the disease in human CMs.

The hiPSC-CMs technique provides multiple advantages, including an unlimited supply of human CMs. Obtaining patient-specific hiPSC-CMs allows for a more personalized medicine and assessment of the genotype-phenotype association, by characterizing the effect of a single-point mutation in a patient, while conserving all its genetic defects and genetic background [17]. The cardiac differentiation of hiPSC into CMs is induced by applying in vitro a specific growth factor, to mimic the signaling pathway involved in the cardiomyogenesis during the early phases of embryogenesis. This leads to the formation of either pacemaker, ventricular or atrial-like CMs, depending on the application of various cytokines concentration combinations and on time-dependent schemes. The cardiac cell fate seems to be controlled in the hiPSC, as it is in embryos.

Albeit these great advantages, the hiPSC-CMs face many limitations. The most challenging one is that represented by the degree of maturity. The ventricular-like CMs differentiated from the hiPSC have a neonatal phenotype that includes some kind of metabolic, structural, functional, and electrophysiological immaturity. The lack of T-tubules and of a normal ultrastructure affect the distribution of the RyR2 and influence calcium homeostasis, which is slower in the hiPSC-CMs as compared to that of adult CMs [115].

Studies in the field develop efforts to improve this maturity. In an attempt to overcome these obstacles, several groups have engineered methods to further mature the hiPSC-CMs by using techniques which include some electrical and mechanical approaches. These are known as environmental manipulations, three-dimensional approaches and biochemical approaches [116].

The environmental manipulations consist of targeting the electrical and mechanical properties of the hiPSC-CMs. The electrical stimulation improves the calcium handling, increases the ultrastructure organization of the myofibrils and conduction velocity [117]. A specific substrate with grooves promotes the cell alignment, sarcomere orientation and bipolar localizations of the gap junctions to a level close to the in vivo arrangement [118]. Cyclic stretch in combination with electrical pacing lead to a more mature phenotype by increasing cell alignment, contractility, and cell size [119]. However, the mechanism by which the electrical and the mechanical stimulation induce hiPSC-CMs maturation is not fully understood. Interestingly, a recent study has subjected the early differentiated hiPSC-CMs (12 days old hiPSC-CMs, following immediately the first spontaneous cardiac contractions) to electromechanical conditioning. At 28 days of culture, the hiPSC-CMs exhibit adult-like phenotype including the cardiac gene expressions, the mitochondria density, the sarcomere length, the presence of the T-tubules, well organized ultrastructure, oxidative metabolism and positive force/frequency relationship [120, 121]. The combination of cell differentiation and electrical field conditioning results in atrial and ventricular tissues with specific drug responses and gene expression [122].

The three-dimensional (3D) approaches including 3D culture systems, known as organoids, improve the transcriptomic and the metabolic maturation and offer a more accurate model for disease modeling and drug testing by representing the in vivo morphology of the heart [123]. Engineered heart tissue displays greater maturation compared to 2D cultures, as shown by the expression of cardiac markers, better alignment of the cells and developed T-tubules [124]. Despite their great interest, the maintenance of a pure CM population and the provision for the adequate exposure to oxygen and nutrients in the 3D approaches, are challenging. In addition, the 3D approaches are subjected to many disadvantages. For example, disease modeling requires the use of single cells to characterize the diseased phenotype. These single cells are required due to their potential clinical use. Finally, the dose used for drug testing may not be accurate in the organoids since the cell numbers and the size of aggregates are not optimized yet.

Biochemical approaches involve the addition of growth factors, a change in culture medium and in hiPSC-CMs culture duration. For example, the Matrigel mattress improves the contractility in a comparable state of the adult rabbit ventricular CMs and increases both the expression of cardiac markers and sarcomere lengths [125]. Specific cell culture differentiation protocols, such as the differentiation in serum-albumin-free basal medium or simple serum with minimal signaling pathway factors, improve both the structural and physiological maturation properties. Their co-culture with mesenchymal stem cells, which secrete key soluble factors, enhances the differentiation and maturation by increasing the gap junctions, the energy production as well as the structural alignment of hiPSC-CMs [126]. Maturating by a medium enriched in T3 (triiodothyronine) hormone, which is crucial for normal cardiac development through the regulation of isoform-switching at the perinatal period, leads to the extension of the T-tubules network, increases the sarcomere length and the cell size, to further improve the contractility [127]. The combination of T3 and/or dexamethasone with the Matrigel mattress (extracellular matrix with physiological stiffness), induces the ventricle-like ECC characterized by T-tubules development, a uniform Ca^2+^ release, a promoted RyR2 structural organization and enhanced CICR mechanism [128]. The long-term culture (of up to 360 days) induces the formation of the sarcomeric M-band, a hallmark of structural sarcomeric maturation, yet, it remains as a quite unpractical method [129]. Some studies even applied a culture media supplied with lactose and deprived of glucose to reach a more mature metabolic activity towards the oxidative metabolism, which also improved the electrophysiological parameters and increased the sarcomere length [130]. Recently, we demonstrated that the low oxygen exposure during the first stage of 3D-cardiac differentiation is essential to enhance the contractile force and to promote a better Ca^2+^ handling properties [131]. These approaches improved the maturity of hiPSC-CMs. However, since they did not reach the adult-like state; and despite as described some methods for inducing more T-tubule maturation, most laboratories struggle and fail to obtain a physiological control of calcium physiology in hiPSC-CMs; the improvement of maturation techniques is still required.

## Cardiac ryanopathies harbored by hiPSC-CMs

### CPVT syndrome and hiPSC-CMs

Investigating the basic underlying pathological mechanisms leading to CPVT has faced difficulties and controversies due to the limited models available. Fatima et al. were the first to model the CPVT syndrome, using patient-specific hiPSC-CMs. In this work, they characterized a novel RyR2 heterozygous mutation, the p.F2483I-, associated with the CPVT syndrome. This mutation induced spontaneous Ca^2+^ events that led eventually to DAD and arrhythmias after catecholaminergic stimulations [132].

Other studies have used hiPSC-CMs to characterize novel heterozygous mutations associated with the CPVT syndrome. These are summarized in Table 2. The use of the hiPSC model as a tool for drug screening was emphasized in 2012 by Itzaki and coworkers. They found that the Thapsigargin treatment,—a specific inhibitor of the SERCA2A—, and that the Flecainide,—a sodium channel blocker—, were effective to abolish the abnormal release of Ca^2+^ in patient-specific CPVT hiPSC-CMs [133]. Another group reported, for the first time, some electrophysiological defects characterized by the occurrence of early and delayed afterdepolarizations,—a key marker of the pathogenesis of the CPVT syndrome—. An abnormal intracellular Ca^2+^ cycling, reduced SR Ca^2+^ load and SR Ca^2+^ leak was also reported in RyR2-P2328S hiPSC-CMs [134]. A group showed abnormal Ca^2+^ transients and DADs responsible for the arrhythmic phenotype in 3D beating CPVT hiPSC-CMs clusters, under both stress and resting conditions. The CaMKII inhibition rescued the cardiac function by reducing the DADs in CPVT hiPSC-CMs [135]. Using specific compounds and genome editing, the critical roles of CaMKII-dependent re-entry and CaMKII-dependent phosphorylation at Ser2814 in RyR2 were shown to be deleterious for the first time in the two human engineered tissue models of CPVT, harboring the RyR2-D358N and RyR2-R4651I mutations, respectively. Adrenergic stimulation and quick electrical stimulation, but not resting condition, caused re-entrant rhythms in the CPVT tissues [136]. We demonstrated that the RyR2-D3638A mutant channels cause, not only a SR Ca^2+^ leak and some impaired contractile properties under stress conditions, but also some post-translational modifications including the FKBP12.6 (FKBP12.6) depletion from the RyR2 macromolecular complex in patient-specific hiPSC-CMs [137]. This particular patient was resistant to the β-blocker Metoprolol treatment but not to Flecainide. The Metoprolol resistance observed in the proband was also revealed in the patient-specific hiPSC-CMs, which highlights the interest in hiPSC-CMs for channelopathy modeling and personalized medicine [137]. Table 2 summarizes the RyR2 mutations associated with CPVT1 syndrome that were investigated using hiPSC-CMs. The localization of the mutations on the RyR2 structural domains is displayed.

**Table 2:** List of CPVT1 syndromes modeled using the hiPSC-CMs.

Localization	Mutations	Findings	Ref.
	D358N	CPVT tissues display re-entrant rhythms under stress that are prevented by CaMKII inhibition.	[60]
N-terminal domain	S406L	The β-adrenergic stimulation by isoproterenol induced DADs and diastolic Ca^2+^ leak, that were reduced with the Dantrolene treatment.	[61]
	E2311D/Q231D	Increased spontaneous calcium sparks and DADs, that were normalized by a CaMKII inhibition.	[62]
	R420Q	Non-ionotropic and lusitropic effects, increased arrhythmias and intracellular Ca^2+^ associated with immature ultrastructural features.	[63]
	ΔExon 3	Dantrolene treatment reduced the premature ventricular complexes and the abnormal Ca^2+^ release in 4 CPVT patients and CPVT hiPSC-CMs. However, Dantrolene was not effective to treat patients carrying mutations in or near the transmembrane domain of the RyR2.	[64]
Helical domain 1	F2483I	The reduction of Ca^2+^ stores induced by a higher CICR mechanism led to an abnormal Ca^2+^ homeostasis. These abnormalities were verified in 2018 in gene-edited CPVT hiPSC-CMs generated by the CRISPR/Cas9 technology.	[65–67]
	P2328S	The abnormal calcium homeostasis and the reduction of the SR Ca^2+^ load led to EADs and DADs at baseline and under isoproterenol stimulation. Another study found that the CPVT hiPSC-CMs exhibit increased non-alternating variability of Ca^2+^ transients and slow depolarization under isoproterenol stimulation.	[68, 69]
	P2328S, T2538R	See findings of the ΔExon 3 mutation.	[64]
	Y2476D	Arrhythmic events and impairment of the calcium handling and beating properties of CPVT hiPSC-CMs. These abnormalities were more pronounced under β-adrenergic stress.	[70]
Central domain	M4109R	The β-adrenergic stimulation induces DADs and irregular Ca^2+^ transients that were abolished with the Flecainide and Thapsigargin treatments.	[71]
	L4115F, Q4201R	See findings of the ΔExon 3 mutation.	[64]
	L3741P	The Flecainide treatment abolished the DADs and the spontaneous calcium sparks.	[72]
	D3638A	The RyR2 macromolecular complex remodeling, including FKBP12.6 depletion, SR Ca^2+^ leak and impaired contractile properties were observed in RyR2-D3638A hiPSC-CMs under stress conditions. Abnormal release of Ca^2+^ were prevented with the Flecainide and S107 treatments but not with the Metoprolol.	[73]
	R4651I	CPVT tissues display re-entrant rhythms under stress that are prevented by CaMKII inhibition.	[60]
Channel domain	V4653F	See findings of the P2328S mutation.	[64]
	I4587V	DADs and abnormal diastolic Ca^2+^ release were observed under β-adrenergic stress. The S107 treatment reduced the occurrence of DADs.	[74]
	R4959Q	See findings of the Y2476D mutation.	[70]

In all these studies, the CPVT hiPSC-CMs were generated from patients harboring RyR2 mutations. Modeling of monogenic arrhythmias through hiPSC-CMs are a pertinent tool to provide insights for risk prediction, pathophysiological mechanisms and the discovery of potential new drugs.

Moreover, the hiPSC model was used to characterize a mutation in the *TERCL* gene leading to the CPVT syndrome. Higher diastolic Ca^2+^ concentration, lower SR Ca^2+^ load due to decreased SERCA and NCX activity, smaller Ca^2+^ transient amplitude and prolonged APs were observed in homozygous TERCL-hiPSC-CMs [138]. Prolonged APs were verified in TERCL knockdown hESC-CMs. However, the Flecainide treatment was effective to reduce the triggering activity of the TERCL-hiPSC-CMs.

The CRISPR/Cas9 technology was used to introduce 3 different CPVT1 mutations, the RyR2-R420Q, the RyR2-Q4201R and the RyR2-F2484I in hiPSC-CMs. These mutant hiPSC-CMs exhibited aberrant Ca^2+^ releases and irregular Ca^2+^ sparks. In contrast to RyR2-R420Q, the RyR2-Q4201R and the RyR2-F2484I hiPSC-CMs exhibited large SR Ca^2+^ leak and decreased SR Ca^2+^ content. These abnormalities were effectively supressed by the Rycal compound, the JTV519, which was more effective than Flecainide and dantrolene. These results suggested mutation site-specific effect on drug responsiveness and Ca^2+^ signaling abnormalities [139].

It should be noted that immature hiPSC-CMs that lack T-tubules are not a very appropriate model for CPVT and the use of these cells to model arrhythmogenic disorders is quite challenging. However, by using patient-derived CMs and the exciting CRISPR/Cas9 technology allowing genome editing, we are now able to generate isogenic CMs that differ only at the single RyR2 variant. The phenotypes for the mutant patient-derived hiPSC-CMs is perhaps the best evidence yet for a causal link for a RyR2 variant and inherited cardiac arrhythmias. Thus, such combined innovative biotechnologies definitely support the hiPSC for disease modeling.

### SC-PMVT syndrome and hiPSC-CMs

The consequences of RyR2-associated mutation with the SC-PMVT at rest are unclear. Our group was the first who revealed the effect of RyR2-H29D mutation associated with PMVT syndrome, a CPVT-like phenotype, at rest using a human cardiac model [140]. The RyR2-H29D mutation causes a diastolic SR Ca2^+^ leak, abnormal mechanical and electrical properties such as AP shortening and DADs and post-translational modification of the RyR2 macromolecular complex including PKA-phosphorylation, S-nitrosylation, oxidation and depletion of FKBP12.6 under non stress conditions. These abnormalities were completely abolished when the RyR2-H29D mutation was reverted using CRISPR/Cas9 technology [140].

## Conclusions

**Fig. 3:**
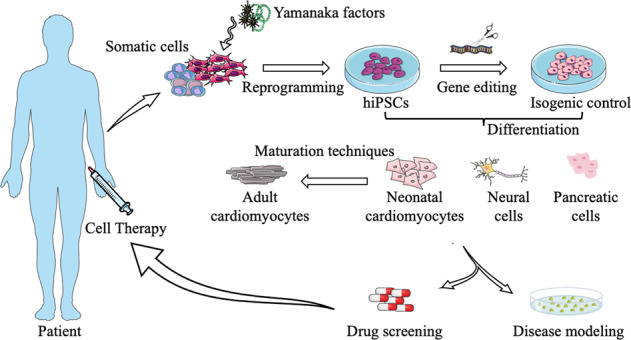
Potential applications of patient-specific induced pluripotent stem cells for patients harboring ryanopathies. Recapitulative scheme illustrating the potential use of hiPSC generated from a patient blood sample or somatic cells and carrying RyR2 mutation. Some isogenic control hiPSC could be generated by correcting the single-RyR2-point mutation using CRISPR/Cas9 technology. The hiPSC could then be differentiated into cardiomyocytes, neural and pancreatic cells. These generated cells could be used for disease modeling and drug screening approaches for a potential patient-specific cell therapy.

To sum up, the RyR2 obviously plays a major part in the pathogenesis of different diseases related to cardiac, neurodegenerative, and metabolic disorders. Calcium homeostasis regulated by the RyR2 is crucial for cell metabolism and it also helps signaling some physiological and pathophysiological conditions. Ryanopathies and RyR2 post-translational modifications lead to a SR Ca^2+^ leak that is responsible for various pathogenic patterns. Finding new tools to model human ryanopathies in the dish and provide for some patient-specific methods is therefore of obvious interest for researchers of the field (Fig. 3).

## Data Availability

Correspondence and requests for materials should be addressed to ACM.
