# Correction: “Ryanopathies” and RyR2 dysfunctions: can we further decipher them using in vitro human disease models?

**DOI:** 10.1038/s41419-022-05468-3

**Published:** 2022-11-30

**Authors:** Yvonne Sleiman, Alain Lacampagne, Albano C. Meli

**Affiliations:** PhyMedExp, University of Montpellier, INSERM, CNRS, Montpellier, France

**Keywords:** Ventricular tachycardia, Ventricular tachycardia

Correction to: *Cell Death and Disease* (2021) 12:1041 10.1038/s41419-021-04337-9, published online 01 November 2021

The original version of this article contained a mistake. During the eproofing procedure, the authors found that the entire 74 references of the 2 tables were not included. All these references should be additionally included in the review. All references can be found below. We apologize for this error (Tables 1 and 2).

**Table 1:** List of the RyR2 mutations associated with the CPVT syndrome.

	Localization	Mutations	Findings	References
Functional characterization of the RyR2 mutants	N-terminal domain	E189D	The RyR2-E189D mutation increased the propensity for SOICR, without altering the FKBP12.6 affinity to bind to the channel.	[1]
		G230C	This novel CPVT mutation enhances RyR2 cytosolic Ca^2+^ sensitivity, which leads to diastolic SR Ca^2+^ leak under stress conditions. RyR2 leak was associated with a depletion of the stabilizing FKBP12.6 protein, which eventually provoked arrhythmias.	[2]
		ΔExon 3	The *RYR2* exon 3 deletion causes an NTD alteration and results in a Ca^2+^ release properties adjustment. Although this deletion is rescued by the β strand switching, it affects interfaces with other *RYR2* domains. This suggests some N-terminal domain and channel pore coupling.	[3]
		G357S	The RyR2-G357S mutation reduced the expression of the RyR2 protein and increased the arrhythmogenic SOICR in HEK293 cells, which might be responsible for the CPVT syndrome.	[4]
		A165D	The RyR2-A165D mutation was first identified in a CPVT patient. When using a knock-in mice model, the A165D mutation induced SR Ca^2+^ release triggering DADs. The A165D mutation was located in the conformational stability loop, which explained the occurrence of some diastolic leak that is responsible for arrhythmias.	[5]
	Helical domain 1	S2246L	Increase of Ca^2+^ release in HL-1 cardiomyocytes expressing mutant hRyR2, after caffeine and β-adrenergic activation.	[6]
		P2328S	This mutation decreases FKBP12.6 binding to the RyR2. Sensitivity increases with cytosolic Ca^2+^ allowing a higher open probability of RyR2 channels at low diastolic levels, causing SR Ca^2+^ leaks in the CPVT1 syndrome. The JTV519 Rycal molecule rescued a normal RyR2 function.	[7]
		R2401H	RyR2-R2401H mutation is located in the FKBP12.6 RyR2 binding region, which could affect the CICR and the ECC resulting in a CPVT.	[8]
		S2246L, R2474S	RyR2 mutations increased both store-overload-induced Ca^2+^ release (SOICR) activity and sensitivity towards luminal calcium, without affecting the channel affinity for the FKBP12.6 in CPVT.	[9]
		N23861	The RyR2-N23861 mutation induced some sensitivity impairment towards Ca^2+^-dependent channel inhibition.	[10]
		R2267H	A novel mutation was identified in sudden infant death syndrome cases. When using some heterologous system expression, this mutation was leaky under beta-adrenergic stimulation, leading to a PKA-phosphorylation that triggers cardiac arrhythmias. Interestingly, another study demonstrated a lack of pathogenicity of this variant. Thus, the in vitro functional findings were not translated to human phenotype.	[11, 12]
		R2474S	The RyR2-R2474S mutation perturbed the interdomain conformational changes, which destabilized the closed state of the RyR2 and lead to a leaky channel.	[13, 14]
	Central domain	N4104K	See findings of the S2246L mutation.	[6]
		Q4201R	See findings of the P2328S mutation.	[7]
		Q4201R	See findings of the S2246L and R2474S mutations.	[9]
		S4153R	This novel RyR2 heterozygous mutation was first described in a 25-year-old CPVT syndrome female patient. This mutation is characterized by some RyR2 gain-of-function that is induced by the SOICR threshold reduction and some propensity increase for spontaneous calcium release.	[15, 16]
	Channel domain	R4497C	See findings of the S2246L mutation.	[6]
		V4653F	See findings of the P2328S mutation.	[7]
		I4867M,	See findings of the S2246L and R2474S mutations.	[9]
		A4860G	When using mice models and HEK293 cells, the RyR2-A4860G mutation reduced the channel activity by inhibiting Ca^2+^ release during the diastole and by overloading the SR with Ca^2+.^ Consequently, it prolonged Ca^2+^ release and corresponding AP, leading to the activation of the NCX exchanger. The I_Ti_ current triggers the early afterdepolarizations (EADs) that are responsible for CPVT pathogenesis.	[17, 18]
		S4565R	Two novel mutations were identified in sudden infant death syndrome cases. When using some heterologous system expression, these 2 mutations were leaky under beta-adrenergic stimulation, leading to a PKA-phosphorylation that triggers cardiac arrhythmias.	[11]
		R4496C (human: R4497C)	The RyR2-R4496C mutation induced an increase in the SR Ca^2+^ load responsible for Ca^2+^ waves and arrhythmias in CPVT murine model.	[19, 20]
		K4750Q	The RyR2-K4750Q mutation mediated-CPVT induced diastolic SR Ca^2+^ leak was caused by an enhancement of propensity to activation of cytosolic and luminal Ca^2+^ and by the loss of cytosolic Ca^2+^/Mg^2+^-mediated inactivation.	[21]
		I4855M	The RyR2-I4855M mutation was present in 2 members of a CPVT-affected family. The RyR2-I4855M shows some loss of function and is characterized by some CICR inhibition of the HEK293 cells. The I4855A may interfere with Ca^2+^ permeation and may affect interactions between the RyR2 pore subunits.	[22]
Case reports and genotyping studies of patient cohorts	N-terminal domain	R414L, I419F, P164S	Novel RyR2 mutations were associated with the CPVT1 syndrome in a swimming-triggered arrhythmia syndrome using direct DNA sequencing and denaturing high-performance liquid chromatography. The 388 unrelated patients were chosen according to family or personal history of drowning or swimming-related cardiac events. However, considering the large number of the cohort, they did not specify the cardiac phenotype of each patient.	[23]
		ΔExon 3, A77V	In a 17-year-old boy postmortem study, the RyR2-A77V mutation was associated with both an arrhythmogenic right ventricular cardiomyopathy and a CPVT syndrome, in the same family. This 17-year-old boy presented right ventricular fibrofatty and fatty myocardium replacement and calcium phosphate deposits in right ventricular cardiomyocytes that were mostly restrained into mitochondria. His mother and his sister presented normal right and left ventricles volume and no kinetic alterations. The exercise treadmill stress test revealed polymorphic ventricular tachycardia that was successfully abolished with β-blocker (Acebutolol) treatment. The same RyR2-A77V mutation led to distinct diseases in the same family members. This reflects the complexity of clinical diagnosis and the variable phenotype that can be present even among family members of the same family. De novo *RYR2* exon 3 deletions were reported in a severe CPVT case. This patient also developed some left ventricular non-compaction (LVNC), which exacerbates the arrhythmia. This patient showed no sign of endomyocardial inflammation and displayed normal heart structure. Multiform premature ventricular contractions, ectopic atrial rhythm, and ventricular triplet was observed during exercise. She experienced ventricular fibrillation and underwent ICD implantation together with the administration of Metoprolol and then Satolol treatment. Due to the severity of her phenotype, she started Flecainide and Nadolol treatment and underwent bilateral sympathectomy. The interaction between RyR2- ΔExon 3 and LVNC that may represent a predictive clinical marker for a more severe CPVT phenotype remains unclear.	[24, 25]
		R414C	The molecular autopsy revealed novel mediated CPVT syndrome RyR2 mutations in 2 unexplained drowning cases. This patient carrying the RyR2-R414C variant experienced syncope and seizure-like symptoms. Unexceptional and unremarkable EEG and physical examination were found. She was first diagnosed with acute seizure activity secondary to trauma. Due to the nature of the sudden death, direct DNA sequencing, and polymerase chain reaction, denaturing high-performance liquid chromatography was performed, which revealed this missense novel RyR2 mutation. As this patient presented a normal structural heart and absence of fatty infiltration, she was considered as a CPVT patient.	[26]
		V186M, P164S	Four patients (3 males) out of 8 patients, were presented with RyR2 mutations associated with some CPVT syndrome. Each patient presented specific symptoms which reflect the heterogeneity of CPVT phenotypes. Some patients had palpitations and seizure-like activity others had a cardiac arrest with ventricular fibrillation. Unfortunately, they did not match each RyR2-variant with its specific phenotype.	[27]
		R169Q	One RyR2 novel heterozygous mutation in exon 8 was screened in an 18-year-old female patient presenting a CPVT syndrome. This patient presented sudden collapse due to exercise and had bidirectional ventricular tachycardia during the exercise stress test. She had a good response to the β-blocker treatment. This same mutation was found recently in three unrelated females. Interestingly, all of these patients presented left ventricular non-compaction cardiomyopathy, and two of them survived sudden cardiac arrest. In vitro, functional analysis of this mutation revealed an increase of the Ca^2+^ fractional release from the SR and a decreased threshold for overload-induced Ca^2+^ release. It was suggested that this RyR2-R169Q mutation leads to local structural abnormalities within or near the hot-spot regions, which in turn leads to functional perturbations. It leads to allosteric dysregulation by reducing the side chain size and diminishing the positive charge and stacking interaction of the RyR2 protein.	[28–30]
		L62F, M81L, P164S, E243K, F329L, R332W, V377M, G357S, T415R, R420Q, V507I, A549V, S616L, H240R	A cohort of CPVT patients was screened to investigate *RYR2* gene mutations. 34 novel mutations were identified. They did not specify the clinical phenotype of the 155 unrelated patients examined in this study. Interestingly, they proposed a novel targeted genetic testing for CPVT syndrome. They emphasized also the genotype/phenotype relationship as the majority of these mutations were localized in the so-called hot-spot regions.	[31]
		D242V, E243K	The long-term follow-up of 101 CPVT patients showed high cardiac events, despite some β-blockers treatment in 21% of patients with 13% of fatal or near-fatal events. Some of these patients survived cardiac arrest and presented palpitations and syncope accompanied or not with seizures. 80% of these patients were treated with β-blockers (mostly with Nadolol but also with Propranolol, Bisoprolol, Acebutolol, and Pindolol). ICD implantation and Verapamil were added to some patients after the 1^st^ cardiac event. Even though β-blockers lower the cardiac events rate, they are not sufficient alone to prevent arrhythmias.	[32]
		R169L	This mutation was identified in an 8 years-old boy with CPVT and Left Ventricular Hypertrophy. This boy presented with two episodes of emotion-triggered syncope and could not survive the third one, which led to sudden cardiac death. This patient carried two other mutations, the G1339 variant in ATP-binding cassette, sub-family C member 9 (*ABCC9*), and the R52H variant in Potassium Inwardly Rectifying Channel Subfamily J Member 5 (*KCNJ5*). These 2 variants have unknown significance. The combination of CPVT and Left Ventricular Hypertrophy might lead to a more severe fatal phenotype. However, more studies are needed to elucidate the pathophysiological mechanism underlying the structural alterations of this RyR2 mutation. This same mutation was also reported in another 9 years-old girls who experienced a syncopal episode. The ECG findings were not reported.	[33, 34]
	SPRY1	R739H	See findings of the L62F mutation.	[31]
	P1	R1013Q, R1051P	See findings of the L62F mutation.	[31]
	SPRY2	A1136V, T1107M,	See findings of the L62F mutation.	[31]
	Handle domain	E1724K	Independently of the localization of the RyR2 mutations, all CPVT patients presented some bradycardia and responded to the β-blockers (Nadolol, Propranolol, and Metoprolol) treatment. These patients presented mono or polymorphic premature ventricular beats (MPVB/PPVB) that trigger bidirectional ventricular tachycardia and polymorphic ventricular tachycardia (PMVT) salvos. 9-year-old was the median age of symptoms onset. The proband carrying the RyR2-E1724K mutation presented monomorphic bigeminy (BG) and PMVT upon exercise stress test.	[35]
		E1837K, E2045G	See findings of the L62F mutation.	[31]
		V1810L	A novel CPVT syndrome-associated RyR2 mutation was identified during the screening of 35 Kazakhstani patients. This low-penetrance variant was found in a 42-year-old Korean proband. Initially, this patient was diagnosed with idiopathic arrhythmia characterized by unstable paroxysms of ventricular tachycardia. He presented bigeminy with a sinus rate of 83 bpm and reached 220 bpm during VT, which was monomorphic.	[36]
	Helical domain 1	S2246L, R2474S	Priori’s group was the first who reported a direct relationship between RyR2 missense variants and CPVT syndrome. 4 missense mutations have been identified, including 3 de novo. The RyR2-S2246L variant was identified in an 8-year-old boy who presented spontaneous onset of bidirectional VT upon isoproterenol infusion. Nadolol and ICD implantation proved effective for this proband. The RyR2-R2474S variant was also found in an 8-year-old boy who presented non-sustained bidirectional VT upon exercise stress test. He was treated with Atenolol.	[37, 38]
		P2328S	Missense RyR2 gene mutation was identified in CPVT patients, which could affect myocardial calcium signaling.	[39]
		R2311D, E2311D	The arrhythmogenic events occurred in young RyR2 mutations-affected patients when compared to ungenotyped CPVT patients, with a higher risk of syncope for males.	[40]
		V2306I, P2328S	Novel mutations were found to be associated with the CPVT syndrome in 12 Finnish probands.	[41]
		A2387P	Novel RyR2 mutation was screened and identified using the DHPLC approach.	[42]
		A2403T	See findings of the R414L mutation.	[23]
		L2487I	RyR2 mutation was detected in 6% of unrelated genotype-negative and atypical LQTS, that were considered CPVT patients.	[43]
		A2254V, A2394G	Independently of the localization of the RyR2 mutations, all CPVT patients presented some bradycardia and responded to the β-blockers treatment. 9-year-old was the median age of symptoms onset. The proband carrying the RyR2-A2254V mutation survived cardiac arrest (CA) and presented BG and polymorphic couplets (PC) upon exercise stress test. Whereas, the patient carrying the RyR2-A2394G mutation presented with seizures during the syncopal events and survived CA. Her exercise stress test revealed MPVB and PMVT.	[35]
		V2475F	The molecular autopsy revealed novel mediated CPVT syndrome RyR2 mutations in 2 unexplained drowning cases. The boy had negative toxicology screen results and no sign of trauma and structural cardiovascular abnormalities. Direct DNA sequencing revealed the presence of this novel RyR2-V2475F variant.	[26]
		R2359Q	Novel RyR2 mutations were identified in 2 CPVT families. The ECG performed for 3 patients from these families, revealed U-wave alterations	[44]
		L2534V	A 13-year-old boy case study, with some novel RyR2 heterozygous mutation. An implantable recording loop was used to diagnose arrhythmogenic disorders.	[45]
		R2404T	Some RyR2 novel heterozygous mutations were shown to be associated with a CPVT syndrome, in a family exhibiting some long QT syndrome.	[46]
		F2307L	Genetic screening for long QT and CPVT syndrome patients in Norway.	[47]
		V2113M, Y2156C, H2168Q, E2183V, D2216V, E2296Q, F2307L, V2321M, R2404T, R2420W, M2389L	See findings of the L62F mutation.	[31]
		H2217Y, C2402Y	See findings of the D242V mutation.	[32]
		G2337V	The β-blockers treatment suppressed severe arrhythmias in stress-induced CPVT-related RyR2 mutations, though it did not prevent the less severe ones.	[48]
		L2527W	Determination of a novel RyR2 heterozygous mutation in a 9-year-old Chinese boy, misdiagnosed with epilepsy and CPVT syndrome. The β-blocker (Metoprolol) treatment proved unfavorable.	[49]
		E2296K	This RyR2-E2296K mutation was identified in a 5-year-old Chinese boy with CPVT using whole exome sequencing. This mutation might reduce protein stability. However, further investigations are needed to prove its causality.	[50]
		V2193L	The RyR2-V2193L mutation was identified in a 9-year-old Chinese boy who presented with both epilepsy and CPVT syndrome. The exercise stress test revealed frequent PPVB and PMVT with the presence of R on T. His electroencephalogram (EEG) showed frequent epileptiform discharges during stage II, stage III, and REM sleep. He was successfully treated with Metoprolol and Levetiracetam.	[51]
		C2277R	The RyR2-C2277R variant, located in the calstabin-binding domain, was identified in 8 members of the same family. The proband and her other family members presented ventricular extrasystoles (VE), bigeminy and/or trigeminy, doublets, and non-sustained VT upon exercise stress test and adrenaline test. These patients showed similar responses but different ventricular arrhythmias complexity degrees. The proband was treated with a combination of ICD implantation, Flecainide, and Nadolol. The other family members were treated either with Atenolol, Nadolol, or with the combination of Nadolol and Flecainide or Atenolol and Flecainide, which proved effective.	[52]
		G3037D	Identification of a novel RyR2 heterozygous mutation in a 2 years old patient exhibiting some CPVT syndrome.	[53]
	Helical domain 2	N4104K	See findings of the mutation S2246L. The RyR2-N4104K variant was identified in a 14-year-old boy who presented non-sustained bidirectional VT upon exercise stress test. This proband was efficiently treated with Atenolol.	[37, 38]
	Central domain	Q4201R	Missense RyR2 gene mutation was identified in CPVT patients, which could affect myocardial calcium signaling.	[39]
		L3778F, G3946S	See findings of the R2311D mutation.	[40, 54]
		N4097S, E4146K, T4158P	In a postmortem genetic testing model, 3 novel mutations were identified in 7 cases of sudden unexplained death, that might potentially cause CPVT.	[55]
		F4020L, E4076K, N4104I, H4108N, H4108Q	Independently of the localization of the RyR2 mutations, all CPVT patients presented some bradycardia and responded to the β-blockers treatment. 9-year-old was the median age of symptoms onset. The proband carrying the RyR2-F4020L mutation presented with seizures during the syncopal events. His exercise stress test revealed BG, PC, and PMVT. Unfortunately, he died suddenly at the age of 20. The proband carrying the RyR2-E4076K mutation presented BG and PMVT upon exercise stress test. The patient carrying the RyR2-N4104I mutation presented with seizures during the syncopal events. His exercise stress test revealed PPVB and sustained PMVT. The proband carrying the RyR2-H4108N mutation survived CA and presented BG, PC, and PMVT upon exercise stress test. Whereas, the patient carrying the RyR2-H4108Q mutation presented MPVB and PMVT upon exercise stress test. The symptoms of these patients reflect the complexity and variability of the clinical phenotype of CPVT patients, which allowed the assessment of a genotype-phenotype correlation.	[35]
		S3938R, T4196A,	See findings of the V186M mutation.	[27]
		L4105F	Novel mutation of the RyR2 mediated CPVT syndrome in 21 years old male. A β-blocker (Metoprolol) and calcium channel blocker (Verapamil) treatment, combined with the successful placement of a dual-chamber implantable cardioverter defibrillator, proved effective.	[56]
		R4144C	See findings of the F2307L mutation.	[47]
		L3879P, Q3925E, G3946A, S3959L, M3972I, D3973H, L3974Q, K3997E, S4124G, Y4149s, R4157Q, Q4159P, N4178S, E4187Q	See findings of the L62F mutation.	[31]
		S3799P, G3946D, D3977Y, A4091V, A4091T	See findings of the D242V mutation.	[32]
		F4174L	A novel heterozygous mutation of the *RYR2* gene associated with CPVT syndrome was identified in a 17-year-old Caucasian boy. Interestingly, arrhythmias had occurred both at rest and under sympatho-adrenergic stimulation conditions.	[57]
		A4282V, R4307C, G4315E	See findings of the L62F mutation.	[31]
	Unspecified domain	K4392R	Case report of an athlete woman harboring some gain-of-function RyR2-K4392R mutation associated CPVT syndrome.	[58]
		R4497C	See findings of the mutation S2246L. The RyR2-R4497C variant was identified in a 30-year-old female who presented non-sustained bidirectional polymorphic VT upon exercise stress test. Two of her sisters died suddenly at the age of 14 and 16, respectively. Variable age-related manifestation of the disease has been thus suggested. This proband was treated with ICD implantation.	[37, 38]
	Channel domain	V4653F	Missense RyR2 gene mutation was identified in CPVT patients, which could affect myocardial calcium signaling.	[39]
		V4771I, A4860G, I4867M, N4895D, E4950K	See findings of the R2311D mutation.	[40]
		P4902L, R4959Q	Three novel mutations were found to be associated with the CPVT syndrome in 12 Finnish probands.	[41]
		N4504I, A4608P, V4880A, M4504I, A4607P	Four novel RyR2 mutations were screened and identified using the DHPLC approach.	[42]
		F4499C, A4510T, G4671R, I4848V	See findings of the R414L mutation.	[23]
		A4556T, 4657-4658EYinsertion, G4671R	RyR2 mutations were detected in 6% of unrelated genotype-negative and atypical LQTS, that were considered CPVT patients.	[43]
		G4662S, H4762P, P4902S	Independently of the localization of the RyR2 mutations, all CPVT patients presented some bradycardia and responded to the β-blockers treatment. 9-year-old was the median age of symptoms onset. The probands carrying the RyR2-G4662S and RyR2-H4762P mutations presented BG and PMVT upon exercise stress test. The patient carrying the RyR2-P4902S presented PPVB and PMVT upon exercise stress test.	[35]
		R4959Q	This mutation was identified in 11 patients of the same family. Four patients were diagnosed with bidirectional tachycardia. Five patients presented monomorphic ventricular tachycardia. Two patients died suddenly while asleep.	[59]
		F4851C, N4895D	Two novel RyR2 mutations were identified in 2 CPVT families. The ECG performed for 3 patients from these families, revealed U-wave alterations	[44]
		F4511L	See findings of the R2404T mutation.	[46]
		E4431K, E4611K	Genetic screening for long QT and CPVT syndrome patients in Norwegia.	[47]
		S4565R, E4611K, W4645R, K4650E, N4736 Del, R4790Q, K4805R, R4822H, G4936R	See findings of the L62F mutation.	[31]
		F4851L	See findings of the D242V mutation.	[32]
		G4671V	See findings of the G2337V mutation.	[48]
		D4631V	A novel CPVT syndrome-associated RyR2 mutation was identified during the screening of 35 Kazakhstani patients. This de-novo missense variant was identified in a 23-year-old female Kazakh. 13-year-old was the age of symptom onset. She experienced syncopal episodes and MPVB/PPVB that trigger bidirectional ventricular tachycardia and PMVT salvos. Since childhood, this patient suffered from dizziness, frequent respiratory infections, scoliosis, palpitation, and chronic pyelonephritis along with the CPVT syndrome. She underwent ICD implantation together with the administration of β-blockers treatment.	[36]

**Table 2:** List of CPVT1 syndromes modeled using the hiPSC-CMs.

Localization	Mutations	Findings	References
	D358N	CPVT tissues display re-entrant rhythms under stress that are prevented by CaMKII inhibition.	[60]
N-terminal domain	S406L	The β-adrenergic stimulation by isoproterenol induced DADs and diastolic Ca^2+^ leak, which were reduced with the Dantrolene treatment.	[61]
	E2311D/ Q231D	Increased spontaneous calcium sparks and DADs, that were normalized by a CaMKII inhibition.	[62]
	R420Q	Non-ionotropic and lusitropic effects increased arrhythmias and intracellular Ca^2+^ associated with immature ultrastructural features.	[63]
	ΔExon 3	Dantrolene treatment reduced the premature ventricular complexes and the abnormal Ca^2+^ release in 4 CPVT patients and CPVT hiPSC-CMs. However, Dantrolene was not effective to treat patients carrying mutations in or near the transmembrane domain of the RyR2.	[64]
Helical domain 1	F2483I	The reduction of Ca^2+^ stores induced by a higher CICR mechanism led to abnormal Ca^2+^ homeostasis. These abnormalities were verified in 2018 in gene-edited CPVT hiPSC-CMs generated by the CRISPR/Cas9 technology.	[65–67]
	P2328S	The abnormal calcium homeostasis and the reduction of the SR Ca^2+^ load led to EADs and DADs at baseline and under isoproterenol stimulation. Another study found that the CPVT hiPSC-CMs exhibit increased non-alternating variability of Ca^2+^ transients and slow depolarization under isoproterenol stimulation.	[68, 69]
	P2328S, T2538R	See findings of the ΔExon 3 mutation.	[64]
	Y2476D	Arrhythmic events and impairment of the calcium handling and beating properties of CPVT hiPSC-CMs. These abnormalities were more pronounced under β-adrenergic stress.	[70]
Central domain	M4109R	The β-adrenergic stimulation induces DADs and irregular Ca^2+^ transients that were abolished with the Flecainide and Thapsigargin treatments.	[71]
	L4115F, Q4201R	See findings of the ΔExon 3 mutation.	[64]
	L3741P	The Flecainide treatment abolished the DADs and the spontaneous calcium sparks.	[72]
	D3638A	The RyR2 macromolecular complex remodeling, including FKBP12.6 depletion, SR Ca^2+^ leak, and impaired contractile properties, were observed in RyR2-D3638A hiPSC-CMs under stress conditions. Abnormal release of Ca^2+^ was prevented with the Flecainide and S107 treatments but not with the Metoprolol.	[73]
	R4651I	CPVT tissues display re-entrant rhythms under stress that are prevented by CaMKII inhibition.	[60]
Channel domain	V4653F	See findings of the P2328S mutation.	[64]
	I4587V	DADs and abnormal diastolic Ca^2+^ release were observed under β-adrenergic stress. The S107 treatment reduced the occurrence of DADs.	[74]
	R4959Q	See findings of the Y2476D mutation.	[70]
